# Obituary

**Published:** 1849-04

**Authors:** 


*Obituary.*—The following letter communicates intelligence of the decease of Dr. A. G. Grow, a graduate of the Medical College in this city, at the last commencement, who is remembered as a young gentleman of great moral worth and promising attainments.

DR. FLINT, CARLTON, MARCH 20TH, 1849.

*Dear Sir:*—My esteemed friend and classmate, Dr. A. G. Grow, is no more. He died in Oakley, La. on the 19th of January last, of cholera, aged 28 years.

He possessed rare qualities of mind, which were peculiarly adapted to the duties and responsibilities of the profession he had chosen; but, alas! he was permitted to enjoy but a brief realization of the labors of our time-honored profession, ere his hopes and aspirations were crushed, and the desires of a benevolent heart quenched by the grave.

I remain, Sir, your Ob't. Serv't.

N. E. BALLOU, M. D.

